# Validity and reliability of the Child Perceptions Questionnaires applied in Brazilian children

**DOI:** 10.1186/1472-6831-9-13

**Published:** 2009-05-18

**Authors:** Taís S Barbosa, Maria Claudia M Tureli, Maria Beatriz D Gavião

**Affiliations:** 1 Department of Pediatric Dentistry, Piracicaba Dental School, State University of Campinas, Piracicaba SP, Brazil

## Abstract

**Background:**

The Child Perceptions Questionnaires (CPQ_8–10 _and CPQ_11–14_) are indicators of child oral health-related quality of life. The aim of this study was to assess the validity and reliability of the self-applied CPQ_8–10 _and CPQ_11–14 _in Brazilian children, after translations and cultural adaptations in the Brazilian Portuguese language.

**Methods:**

Schoolchildren were recruited from general populations for pre-testing (n = 80), validity (n = 210), and test-retest reliability (n = 50) studies. They were also examined for dental caries, gingivitis, fluorosis, and malocclusion.

**Results:**

Children with greater dental caries experience in primary dentition had higher impacts on CPQ domains. Girls had higher scores for CPQ_8–10 _domains than boys. Mean CPQ_11–14 _scores were highest for 11-year-old children and lowest for 14-year-old children. Construct validity was supported by significant associations between the CPQ_8–10 _and CPQ_11–14 _scores and the global rating of oral health (r = 0.38, r = 0.43) and overall well-being (r = 0.39, r = 0.60), respectively. The Cronbach's alpha was 0.95 for both questionnaires. The test-retest reliabilities of the overall CPQ_8–10 _and CPQ_11–14 _scores were both excellent (ICC = 0.96, ICC = 0.92).

**Conclusion:**

The Brazilian Portuguese version of CPQ_8–10 _and CPQ_11–14 _was valuable and reliable for use in the Brazilian child population, although discriminant validity was sporadic due to the fact that impacts are mediated by others factors, such personal, social, and environmental variables.

## Background

Over the past two decades, there has been substantial development of oral health-related quality of life (OHRQoL) assessments [1]. In this context, children have been considered, since they are affected by numerous oral and orofacial disorders, all of which have the potential to impact physical functioning and psychosocial well-being [2-4]. Consequently, the Child Perceptions Questionnaires (CPQ) were developed for measurements, taking into account the children cognitive abilities and lifestyles for an age range from 8 to 10 years (CPQ_8–10_) [5] and from 11 to 14 years (CPQ_11–14_) [6]. These questionnaires assess children's perceptions of the impact of oral disorders on physical and psychosocial functioning.

The need to test the psychometric properties of instruments, such as those for measuring OHRQoL in a new environment, has been stressed [1]. The linguistic and cultural context in which a measure is used can have a bearing on the validity, as can the intended purpose of the measure. Preliminary studies have confirmed the validity and reliability of CPQ_11–14 _in other countries, such as the United Kingdom [7,8], New Zealand [3], Saudi Arabia [9], China [10,11], Australia [12], and Brazil [13], whereas the CPQ_8–10 _has been confirmed in Northern Ireland [14] and Australia [12].

The CPQ_8–10 _has not been validated for use in Brazil or for use in children with varying levels of disease. On the other hand, Goursand et al. [13] demonstrated that the CPQ_11–14 _is applicable to Brazilian children who were receiving dental care, but these authors suggested that the respective psychometric properties should be evaluated in a population study using self-applied questionnaires [13], as considered also by Brown and Al-Khayal [9]. Therefore, the application of the CPQ_8–10 _and CPQ_11–14 _in children with different types and levels of oral diseases from a general population can justify the present study, which aimed to assess the validity and reliability of the CPQ_8–10 _and CPQ_11–14 _in children from Piracicaba city, São Paulo, Brazil, after translations and cultural adaptations in the Brazilian Portuguese language.

## Methods

The questionnaires chosen were developed by Jokovic et al. [5,6] for use as an outcome measure in clinical trials and evaluation studies. The process of crosscultural adaptation and validation consisted of two main steps: a preliminary and a main study. The research project was submitted to and approved by the Research Ethics Committee (No. 021/2006) of the Piracicaba Dental School, State University of Campinas. The children's and parents'/guardians' consent was obtained.

### Preliminary study

The screening process for crosscultural adaptation was conducted according to Guillemin et al. [15] (Appendix). Firstly, two pediatric dentists, fluent in the English and Brazilian Portuguese languages, translated the questions. A conceptual, nonliteral translation was emphasized. The first author (TSB) compared the versions and discussed with translators about the divergences that were found, and a first Portuguese version was achieved. Then, two native English speakers, unaware of the objectives of the study, did a back-translation into English. Next, a committee review, constituting three dentist researchers, and the first author (TSB) compared the source and final versions, solving discrepancies and considering crosscultural equivalence, thus reaching the second version.

#### Pretest technique

For evaluating the language used in the instruments and the structure adaptation, 40 children who were aged from 8 to 14 years were recruited from general populations that were attending public schools in Piracicaba, SP, Brazil. The questionnaires were applied to groups of 20 children in the respective age ranges (8–10 and 11–14). For this purpose, the alternative "I did not understand" was added to each question for identifying the questions that were not understood by the children. Questions with this alternative item that were chosen by 15% or more of the sample were discussed by the committee, who replaced problematic items by culturally accepted ones. After that, the questionnaires were applied to other groups of 20 children, until the item "I did not understand" had not been chosen in any question by 85% or more of the children.

### Main study

Participants in the main study were 210 children aged 8–14 years (30 of each age group) (Table 1) who did not have systemic and/or mental developmental disorders. They are referred to in this study as the CPQ_8–10 _group (n = 90) and CPQ_11–14 _group (n = 120), respectively. These convenience samples were recruited from general populations that were attending five public schools in Piracicaba.

**Table 1:** Distribution of children in accordance with groups, gender and age in each of the study phases

		Pre-testing study		Validity study		Test-retest reliability study	
		
		*n*	%	*n*	%	*n*	%
CPQ group	8–10	20	25.0	90	43.0	20	40.0
	11–14	60	75.0	120	57.0	30	60.0
Gender	Boy	27	33.8	105	50.0	22	44.0
	Girl	53	66.2	105	50.0	28	56.0
Age	8 yrs	6	7.5	30	14.3	5	10.0
	9 yrs	9	11.25	30	14.3	7	14.0
	10 yrs	5	6.25	30	14.3	8	16.0
	11 yrs	18	22.5	30	14.3	12	24.0
	12 yrs	16	20.0	30	14.3	8	16.0
	13 yrs	14	17.5	30	14.3	5	10.0
	14 yrs	12	15.0	30	14.3	5	10.0

#### Data collection

The children were clinically examined for dental caries, gingivitis, fluorosis, and malocclusion by two examiners, who were calibrated in accordance with the WHO Oral Health Surveys: Basic Methods criteria [16]. All examinations took place at the children's school, out of doors in daylight, but not in direct sunlight. The dmft (sum of decayed, missing, and filled teeth in the primary dentition) and DMFT (sum of decayed, missing, and filled teeth in the permanent dentition) indices were used to assess caries status. The periodontal status assessment criteria were those proposed in the WHO's 1997 oral health survey methods manual [16], employing the Community Periodontal Index (CPI). This index classifies periodontal status based on six index teeth (16, 11, 26, 36, 31, 46) in patients under the age of 20 years. The codes were: 0 = healthy and 1 = bleeding observed directly or by using a mouth mirror, after probing. The presence or absence and severity of dental fluorosis were evaluated using the Dean's index criteria (DI) [17] at the following levels: 0 = normal; 1 = questionable; 2 = very mild; 3 = mild; 4 = moderate; and 5 = severe. The recording is made on the basis of the two teeth that are most affected. Malocclusion was scored using the Dental Aesthetic Index (DAI) [18], which assesses the relative social acceptability of dental appearance by collecting and weighing data on 10 intraoral measurements. This enables each individual to be placed on a dental appearance continuum, ranging from 13 (the most socially acceptable) to 100 (the least acceptable), and orthodontic treatment needs can be prioritized based on the predefined categories of 'minor/none' (scores 13 to 25), 'definite' (26 to 31), 'severe' (32 to 35), or 'handicapping' (36 or more) [18].

Previously, the dental examiners underwent a calibration session, resulting in interexaminer kappa scores of 0.96 for DMFT/dmft, 0.80 for fluorosis, 0.73 for gingivitis, and 0.88 for DAI scores. After a period of 2 weeks, the intraexaminer reliability was verified by conducting replicate examinations in 20 individuals, resulting in a kappa score of 0.95 for DMFT/dmft, 0.81 for gingivitis, 0.80 for fluorosis, and 0.97 for malocclusion.

#### Validated and reliability procedures

Each child completed the age-specific CPQ in the classroom just prior to the dental examination; questions were asked about the frequency of events. Response options for the four domains (symptoms; functional limitations – *e.g*., difficulties with chewing – emotional well-being, and social well-being) and the respective scores were: 'Never' (scoring 0); 'Once or twice' (1); 'Sometimes' (2); 'Often' (3); and 'Everyday' or 'Almost everyday' (4). A high score indicates more negative impacts on child QoL. Fifty randomly selected children, 20 from CPQ_8–10 _and 30 from CPQ_11–14_, were invited to fill out a second copy of the questionnaire two weeks later to assess the test-retest reliability [5,6].

### Data analysis

The total CPQ scores for each participant were calculated by summing the item codes, whereas the subscale scores were obtained by summing the codes for questions within the four health domains. Discriminant validity was assessed by comparing overall and domain scores according to the child's age, child's gender, and the severity of the child's oral conditions. Since the items were scored using an ordinal scale and most of the distributions were asymmetrical, nonparametric statistical procedures, such as Mann-Whitney and Kruskal-Wallis tests, were used (as appropriate) to examine the differences between the means of two categories and three or more categories, respectively. To analyze construct validity, the associations between CPQ scores and the two global indicators were determined, using the Spearman correlation coefficient. Internal consistency was assessed by means of Cronbach's alpha and test-retest reliability by Intraclass Correlation Coefficients (ICCs), calculated by the one-way analysis of variance random effects parallel model [19].

## Results

### Characteristics of Participants

Table 1 presents the characteristics of the pretesting, validity, and reliability study participants in terms of an age-specific CPQ group, gender, and age.

### Pretesting Results

While the CPQ_8–10 _group was able to answer all questions of the questionnaire, CPQ_11–14 _group did not understand some questions, as follows: initially, questions 4 (*"How much does the condition of your teeth, lips, jaws or mouth affect your life overall?"*) and 11 (*"In the past 3 months, because of your teeth, lips, mouth or jaws, how often have you breathed through your mouth?"*) showed an index of "not understand" exceeding 15%. The wording of the questions was changed, and the second Brazilian Portuguese version of CPQ_11–14 _was self-applied on a new sample of 20 children. Only one question (40, *"In the past 3 months, because of your teeth, lips, mouth or jaws, how often have other children made you feel left out?"*) was misunderstood and changed. Thus, the third Brazilian Portuguese version was achieved, applied to other sample of 20 children, and was considered appropriate by more than 95% of this group, totaling 20 and 60 children for evaluating the language and the structure adaptation of CPQ_8–10 _and CPQ_11–14_, respectively.

### Discriminant and Construct Validity

#### Child Perceptions Questionnaire (CPQ_8–10_)

There was a distinct gradient in mean CPQ_8–10 _scores across the categories of caries severity, whereby those in the 'dmft ≥ 3' category had the highest and those in the 'dmft = 0' category had the lowest CPQ_8–10 _score, on average (see Additional file 1). Such a gradient was also observed with respect to the social well-being domain scores, but not as regards to the other three domains. While there was an apparent difference in CPQ_8–10 _scores across the categories of DMFT, it did not quite reach statistical significance (see Additional file 1). Girls had higher CPQ_8–10 _scores overall, as well as higher scores for oral symptoms and emotional and social well-being than boys. Children without gingivitis had CPQ_8–10 _higher scores for the overall and emotional well-being domains. No clear statistically significant gradients were observed with respect to the CPQ_8–10 _scores and the following variables: age, malocclusion, and fluorosis (see Additional file 1).

There were significant positive correlations between CPQ_8–10 _scale scores and global oral health ratings (p < 0.001) and overall well-being (p < 0.001). Significant correlations were also observed between the scores for all subscale scores and both global ratings (Table 2).

**Table 2:** Construct validity rank correlations between CPQ scores and global rating of oral health and overall well-being

	CPQ_8–10 _(n = 90)				CPQ_11–14 _(n = 120)			
	Oral Health		Overall Well-being		Oral Health		Overall Well-being	
	
	r^a^	p^b^	r^a^	p^b^	r^a^	p^b^	r^a^	p^b^

Total scale	0.38	< 0.001	0.39	< 0.001	0.43	< 0.001	0.60	< 0.001
Subscales								
Oral symptoms	0.34	0.001	0.34	< 0.001	0.35	< 0.001	0.46	< 0.001
Functional limitations	0.27	0.008	0.37	< 0.001	0.29	0.001	0.51	< 0.001
Emotional well-being	0.43	< 0.001	0.50	< 0.001	0.41	< 0.001	0.56	< 0.001
Social well-being	0.35	< 0.001	0.38	< 0.001	0.41	< 0.001	0.52	< 0.001

^a ^Spearman's correlation coefficient

^b ^p-value

#### Child Perceptions Questionnaire (CPQ_11–14_)

Children with a greater dmft experience had higher CPQ_11–14 _overall scores, as well as higher scores for oral symptoms and emotional and social well-being. No clear statistically significant gradients were observed in mean CPQ_11–14 _scores across the categories of DMFT, fluorosis, and malocclusion severity (see Additional file 2). There were significant differences among eleven- and fourteen-year-old children in the oral symptoms domain score, with the former being the highest and the latter being the lowest. No clear statistically significant gradients were observed in mean CPQ_11–14 _scores across gingivitis categories. While there was an apparent gender difference in the CPQ_11–14 _score, it was not statistically significant (see Additional file 2).

As an index of construct validity, Spearman's correlation was highly significant at the 0.001 level with both global indicators for the CPQ_11–14 _total scale and all subscales (Table 2).

### CPQ Reliability

Cronbach's alpha for both groups as a whole was 0.95 (Table 3). For the domains of the CPQ_8–10 _and CPQ_11–14 _groups, the coefficient ranged from 0.67 for oral symptoms to 0.92 for social well-being, and from 0.75 for oral symptoms to 0.90 for emotional well-being, respectively, indicating acceptable to good internal consistency reliability.

**Table 3:** CPQ_8–10 _and CPQ_11–14 _Reliability Statistics

	CPQ_8–10_			CPQ_11–14_		
	Number of Items	Cronbach's Alpha (n = 90)	ICC (95% CI)* (n = 20)	Number of Items	Cronbach's Alpha (n = 120)	ICC (95% CI)* (n = 30)

*Total scale*	25	0.95	0.96 (0.89–0.98)	37	0.95	0.92 (0.80–0.96)
*Subscales*						
OS	5	0.67	0.85 (0.38–0.90)	6	0.75	0.84 (0.62–0.93)
FL	5	0.82	0.88 (0.70–0.95)	9	0.81	0.78 (0.48–0.91)
EW	5	0.84	0.94 (0.85–0.97)	9	0.90	0.86 (0.62–0.93)
SW	10	0.92	0.94 (0.86–0.97)	13	0.89	0.95 (0.85–0.97)

OS, oral symptoms; FL, functional limitations; EW, emotional well-being; SW, social well-being

* One-way random effect parallel model

The ICC was 0.96 for the overall CPQ_8–10 _scores, indicating perfect agreement, and for the domains it ranged from 0.85 to 0.94, indicating excellent agreement. For the CPQ_11–14 _group, the ICC for the overall scale was 0.92, indicating substantial agreement. The ICC for the CPQ_11–14 _subscales ranged from 0.78 to 0.95, indicating substantial to perfect test-retest reliability (Table 3).

## Discussion

The CPQ has previously been developed and tested in a clinical convenience sample of children in Canada [5,6]. Every time an instrument is used in a new context or with a different group of individuals, it is necessary to reestablish its psychometric properties. In this study, the CPQ_8–10 _and CPQ_11–14 _were applied to a general sample of schoolchildren (8 to 14 yrs of age) in a country (Brazil) with a different cultural context. Prior to validity and reliability tests, the questionnaires were translated, back-translated, and crossculturally adapted in order to ensure their conceptual and functional equivalences.

The following subheadings discuss the results.

### CPQ Pretesting

At the pretesting stage, children from 8 to 10 years old were able to answer all questions in the questionnaire, whereas in the Jokovic et al. [5] study, 8-yr-old children did not relate to the introductory/transition statement, *"In the past 4 weeks, because of your teeth or mouth..."*, when responding to the questions and required either a simpler format or an interviewer-supervised/administered questionnaire. Moreover, in the present study, the children of the CPQ_11–14 _group did not understand some of the questions and required some of the words to be changed to guarantee their cultural equivalence. A few problems were also encountered in the Arabic translation of CPQ_11–14 _with regard to self-reporting of age, and the questionnaire was considered too long for many of the medically compromised patients [9]. Moreover, it was suspected that quite a few of the children asked their parents for help, which probably influenced the replies [9]. Goursand et al. [13] chose to administer the CPQ_11–14 _as an interview in order to avoid the possibility of children soliciting help from their parents when having difficulty understanding the questions. However, these authors [9,13] also suggested that the effect of different modes of administration on the validity and reliability of the instrument should be evaluated in population studies, as done in the present one, in which the questionnaires were self-applied on a population sample, resulting in satisfactory psychometric properties, irrespective of the mode of administration. Therefore, translating and adapting a questionnaire developed in one country for use in another usually results in some changes in the wording, format, and mode of administration, which have been facilitating the development of a culturally relevant instrument [6,9,20-22], being a strong point of the methodology for use in a different setting.

### CPQ Discriminant and construct validity

When testing discriminant validity, a clear ascending gradient was observed for oral symptoms among children aged 11–14 years, with those aged 11 years being the highest and those aged 14 years being the lowest (see Additional file 2); however, this was not observed for the CPQ_8–10 _group (see Additional file 1). This reflects the fact that children's understanding of oral health and well-being is also affected by age-related experiences [2,21]. During mixed dentition (8–12-yr-olds), children experienced many problems related to natural processes, such as exfoliating primary teeth, dental eruption, or space due to a nonerupted permanent tooth, which simultaneously affect their QoL. On the other hand, these conditions were not reported as important causes of oral impacts in other age groups [23]. After 12 years of age, children will move from a transitional dentition, just as they will have altered their concepts of health and probably also have different expectations [1,24].

While there was an apparent gender difference in the CPQ_11–14 _score, it did not quite reach statistical significance (see Additional file 2). These findings suggested that girls tend to report higher impacts on QoL than boys, on average. However, in the Foster Page et al. [3] study, the mean emotional well-being domain score was higher for girls than for boys. One explanation for these variations is related to the differences in the characteristics of selected samples between the Foster Page et al. [3] and the present studies, and patient and general population samples, respectively.

In CPQ_8–10 _group, girls had higher impacts on all CPQ_8–10 _scores than boys (see Additional file 1). There are no studies in the literature that evaluated differences between genders related to oral impacts on QoL during middle childhood (6–10 yrs). Thus, further research on OHRQoL needs to be conducted using samples of this age group in order to elaborate on the findings reported here. Furthermore, these findings were similar to the results of the CPQ_11–14 _group. However, the difference in the significance between the results of the two groups may be explained by the particularity in the cognitive, emotional, functional, and behavioral characteristics of each age group [24]. This implies that the comparison between the results related to age-specific CPQ groups should be interpreted with caution, since they are heterogeneous in terms of stage of development.

Concerning dental caries experience, it was hypothesized that children with more severe caries would have higher impacts on their QoL, corroborating recent studies [3,4,9,25]. However, only primary dentition showed significant correlation with both CPQ scores (see Additional files 1 and 2). There was an ascending difference between dmft and all CPQ_11–14 _domains, except for functional limitations. Such a gradient was also observed with respect to the CPQ_8–10 _social well-being domain but not with the others. These findings may be explained by the fact that adolescents had experienced untreated disease for longer than the younger participants, also reflecting the health view as a multidimensional concept during early adolescence [26].

Analysis within DMFT was not statistically significant but also provided some evidence to suggest that the CPQ_8–10 _scores were associated with the severity of this clinical condition in an expected direction (see Additional file 1). Furthermore, no clear statistically significant gradient was observed with respect to the CPQ_11–14 _scores and DMFT categories (see Additional file 2). It is known that caries progresses more rapidly in primary teeth than in permanent teeth, supporting the hypothesis that deciduous enamel is more susceptible to caries than permanent enamel [27]. Consequently, although dental caries was relatively prevalent in permanent dentition, it did not affect the child's ability to perform daily activities.

No clear gradients were observed in both mean CPQ scores across the categories of malocclusion severity (see Additional files 1 and 2). The results of other studies conducted to date are equivocal [3,28,29]. While some studies indicated good discriminant validity between children with different levels of malocclusion severity [3,29], others did not [28]. Despite the small sample sizes involved with a handicapping malocclusion, which could be considered as a limitation into this context, the lack of marked difference is also consistent with the contemporary models of disease/disorder and its consequences. The model by Wilson and Cleary [30] indicates that health outcomes experienced by an individual are not determined only by the nature and severity of the disease/disorder but also by the personal and environmental characteristics. Moreover, different meanings of QoL vary between and within groups of individuals [31] according to culture and education [23], contributing for distinct impacts of malocclusion on QoL.

Although CPQ_8–10 _and CPQ_11–14 _scores tended to be lowest for the 'fluorosis ≥ 1' category and highest for children without dental fluorosis, differences were not significant (see Additional files 1 and 2). A potential explanation may be the low disease levels in the sample. However, although the levels of fluorosis were low in the Robinson et al. [4] study, the Ugandan children experienced appreciable impacts on OHRQoL. These contradictory outcomes suggest that cultural norms and expectation influence children's perception of their oral health and its effect on their QoL, as considered, since causal pathways between clinical variables may include individual and environment variables as both moderators and mediators [30].

It was observed that children without gingivitis would have higher CPQ_8–10 _scores (see Additional file 1), contrasting with other studies [2,25]. The following explanations may account for the present findings: the clinical instrument was not performed as a discriminant measure, there was oral disease in the small sample size, or the impacts were mediated by a variety of factors, such as relevance. Moreover, while there was an ascending difference between preadolescents without and with gingivitis, it did not quite reach statistical significance (see Additional file 2). The lack of marked difference may be due to the low disease levels in the sample, which caused immeasurably low levels of impact. Furthermore, the way people feel about their QoL also needs to be considered, since it does not develop in isolation from their existing expectations (that constrain what is relevant) as well as the environment in which the margins of relevance are constructed [31].

Finally, the results of this study suggested that both questionnaires have good construct validity (Table 2). Significant correlations were shown between global rating of oral health and overall well-being and the total scale and all subscales, indicating also that children are able to give psychometrically acceptable accounts concerning their health status and its overall effects on their lives [32].

### CPQ Internal Consistency and Test-retest Reliability

Both questionnaires have acceptable reliability with the internal consistency [33] and test-retest reliability [34] (Table 3). Cronbach's alpha and ICCs found in this study were similar to the results from Canada [5,6]. However, in the Jokovic et al. [6] study, the ICC for the social well-being subscale was low at 0.16, suggesting that children are more likely to experience variability over time in social functioning and experiences than in physical and emotional effects of oral and orofacial conditions. An alternative explanation for these contradictory outcomes is that enjoying contact with people might be an inherently unstable construct to children, which varies with time [35].

In addition, children are, in a sense, 'moving targets' not just because childhood is a period with immense changes in psychosocial awareness but because the children's dental and facial features change rapidly [36]. Furthermore, children's cognitive development varies such that the wording of items, specific dimensions, and their relevance and meaning to children of similar ages can differ, and the changes in a child over time can make repeated measurements difficult to compare [37].

## Conclusion

In conclusion, the Brazilian Portuguese version of both questionnaires showed good construct validity, internal consistency, reliability, and test-retest reliability but demonstrated sporadic discriminative validity. Thus, the relationship between child OHRQoL and clinical indicators should be interpreted with caution, since the inconsistencies found in the relationships between clinical data and OHRQoL may not be due to the psychometric properties of the measures but due to the fact that impacts are mediated by others factors, such personal, social, and environmental variables.

Moreover, given the cross-sectional nature of the data studies, the observed findings could address only the descriptive and discriminative potential of OHRQoL measures in relation to child oral conditions. Therefore, further research is required, as these findings were based on cross-sectional study and convenience samples.

## Competing interests

The authors declare that they have no competing interests.

## Authors' contributions

TSB participated in conception and design of the study, data analysis and interpretation, acquisition of data, and drafting the manuscript. MCMT contributed to the data collection. MBDG participated in the conception and design of the study and critical revision of manuscript. All authors read and approved the final manuscript.

## Appendix

Guideline to preserve equivalence in cross-cultural adaptation of composite health status measures*

### 1. Translation

Produce several translations

Use qualified translators

### 2. Back-translation

Produce as many back-translations as translations

Use appropriate back-translators

### 3. Committee review

Constitute a committee to compare source and final versions

Membership of the committee should be multidisciplinary

Use structured techniques to resolve discrepances

Modify instructors or format, modify/reject inappropriate items

Ensure that the translation is fully comprehensible

Verify cross-cultural equivalence of source and final versions

### 4. Pre-testing

Check for equivalence in source and final versions using a pre-test technique

Either use a probe technique

Or submit the source and final versions to bilingual lay people

### 5. Weighting of scores

Consider adapting the weights of scores to the cultural context

Adapted from Guillemin et al. (2003)

## Pre-publication history

The pre-publication history for this paper can be accessed here:



## Supplementary Material

Additional file 1 **CPQ_8–10 _scores by categories of clinical data**. The data provided represent the statistical analysis of the CPQ_8–10 _scores by child's characteristics and categories of clinical data. Click here for file

Additional file 2 **CPQ_11–14 _scores by categories of clinical data**. The data provided represent the statistical analysis of the CPQ_11–14 _scores by child's characteristics and categories of clinical data. Click here for file
