# Supplementary material for: Validity and reliability of the Child Perceptions Questionnaires applied in Brazilian children
Source: BMC Oral Health. 2009 May 18;9:13. doi: 10.1186/1472-6831-9-13 (PMC2696414; doi:10.1186/1472-6831-9-13)
Supplement: Additional file 1 — CPQ8–10 scores by categories of clinical data. The data provided represent the statistical analysis of the CPQ8–10 scores by child's characteristics and categories of clinical data. [file 1472-6831-9-13-S1.doc]

Table 2. CPQ_8-10_ scores by categories of clinical data

|  |  | CPQ_8-10_ Overall Score | | CPQ_8-10_ Domain Scores | | | | | | | |
| --- | --- | --- | --- | --- | --- | --- | --- | --- | --- | --- | --- |
|  |  |  | | Oral Symptoms | | Functional Limitations | | Emotional Well-being | | Social Well-being | |
|  | n | Median | Mean (SD) | Median | Mean (SD) | Median | Mean (SD) | Median | Mean (SD) | Median | Mean (SD) |
| Gender |  |  |  |  |  |  |  |  |  |  |  |
| Boy | 45 | 12.0 | 20.8 (20.6)** | 7.0 | 7.1 (4.0)* | 2.0 | 4.5 (5.2) | 3.0 | 4.8 (5.1)** | 2.0 | 5.7 (8.9)** |
| Girl | 45 | 25.0 | 30.2 (19.8)** | 8.0 | 8.8 (4.0)* | 4.0 | 5.2 (4.5) | 6.0 | 7.0 (4.8)** | 8.0 | 9.3 (8.6)** |
| Age |  |  |  |  |  |  |  |  |  |  |  |
| 8 | 30 | 16.5 | 24.7 (23.3) | 7.0 | 7.6 (4.3) | 2.0 | 4.6 (5.4) | 3.0 | 5.0 (5.2) | 3.0 | 7.6 (10.5) |
| 9 | 30 | 24.0 | 27.5 (18.2) | 8.5 | 8.7 (3.5) | 5.0 | 5.5 (4.3) | 6.0 | 6.8 (4.9) | 7.0 | 8.2 (7.2) |
| 10 | 30 | 21.0 | 24.3 (20.7) | 7.0 | 7.5 (4.5) | 2.5 | 4.4 (4.9) | 5.0 | 5.8 (5.0) | 4.5 | 6.8 (8.7) |
| Dental caries |  |  |  |  |  |  |  |  |  |  |  |
| DMFT = 0 | 64 | 18.0 | 23.2 (17.7) | 7.0 | 7.6 (3.7) | 3.0 | 4.6 (4.5) | 4.0 | 5.3 (4.5) | 4.0 | 6.6 (7.7) |
| DMFT = 1 | 15 | 24.0 | 30.4 (29.6) | 8.0 | 8.6 (5.2) | 5.0 | 6.2 (6.3) | 6.0 | 7.1 (6.5) | 4.0 | 8.7 (12.8) |
| DMFT ≥ 2 | 11 | 34 | 32.3 (21.7) | 9.0 | 8.9 (4.9) | 4.0 | 4.5 (4.7) | 7.0 | 7.6 (5.6) | 11.0 | 11.2 (8.8) |
| dmft = 0 | 42 | 16.5 | 22.2 (18.0) | 7.0 | 7.2 (4.1) | 3.0 | 4.2 (4.5) | 4.0 | 5.1 (4.9) | 3.5 | 5.7 (7.5)* |
| dmft = 1 or 2 | 31 | 21.0 | 25.6 (18.9) | 8.0 | 8.0 (3.7) | 3.0 | 4.5 (4.4) | 4.0 | 5.6 (4.6) | 6.0 | 7.4 (8.0) |
| dmft ≥ 3 | 17 | 28.0 | 33.5 (27.8) | 10.0 | 9.6 (4.6) | 5.0 | 6.8 (6.2) | 7.0 | 8.3 (5.9) | 10.0 | 12.2 (11.8)* |
| Fluorosis |  |  |  |  |  |  |  |  |  |  |  |
| 0 | 73 | 23.0 | 26.7 (21.8) | 8.0 | 8.1 (4.3) | 4.0 | 5.1 (5.1) | 4.0 | 6.1 (5.4) | 5.0 | 8.1 (9.5) |
| ≥ 1 | 17 | 18.0 | 20.5 (13.8) | 7.0 | 7.4 (3.4) | 3.0 | 3.6 (3.5) | 5.0 | 5.1 (3.6) | 4.0 | 4.8 (4.9) |
| Gingivitis |  |  |  |  |  |  |  |  |  |  |  |
| Absence | 63 | 25.0 | 28.9 (22.9)* | 8.0 | 8.4 (4.5) | 4.0 | 5.3 (5.3) | 5.0 | 6.9 (5.4)** | 5.0 | 8.5 (9.8) |
| Presence | 27 | 16.0 | 17.6 (10.9)* | 7.0 | 6.9 (3.0) | 3.0 | 3.8 (3.7) | 2.0 | 3.4 (3.1)** | 3.0 | 5.3 (5.6) |
| Malocclusion |  |  |  |  |  |  |  |  |  |  |  |
| Minor/none | 50 | 17.0 | 21.7 (17.3) | 8.0 | 7.4 (4.0) | 3.5 | 4.5 (4.3) | 4.0 | 5.3 (4.8) | 3.0 | 5.7 (6.9) |
| Definitive | 13 | 25.0 | 35 (29.8) | 8.0 | 9.6 (4.6) | 5.0 | 6.8 (7.0) | 5.0 | 7.8 (6.4) | 6.0 | 10.8 (13.9) |
| Severe | 19 | 22.0 | 26.7 (17.7) | 7.0 | 8.1 (3.5) | 3.0 | 3.8 (3.7) | 5.0 | 5.9 (4.6) | 7.0 | 8.9 (8.0) |
| Handicapping | 8 | 23.0 | 31.1 (26.4) | 8.0 | 8.5 (5.3) | 5.0 | 6.5 (6.2) | 4.5 | 5.9 (5.7) | 7.0 | 10.3 (10.4) |

*p≤0.05

**p≤0.01
