# Supplementary material for: Validity and reliability of the Child Perceptions Questionnaires applied in Brazilian children
Source: BMC Oral Health. 2009 May 18;9:13. doi: 10.1186/1472-6831-9-13 (PMC2696414; doi:10.1186/1472-6831-9-13)
Supplement: Additional file 2 — CPQ11–14 scores by categories of clinical data. The data provided represent the statistical analysis of the CPQ11–14 scores by child's characteristics and categories of clinical data. [file 1472-6831-9-13-S2.doc]

Table 3. CPQ_11-14_ scores by categories of clinical data

|  |  | CPQ_11-14_ Overall Score | | CPQ_11-14_ Domain Scores | | | | | | | |
| --- | --- | --- | --- | --- | --- | --- | --- | --- | --- | --- | --- |
|  |  |  | | Oral Symptoms | | Functional Limitations | | Emotional Well-being | | Social Well-being | |
|  | n | Median | Mean (SD) | Median | Mean (SD) | Median | Mean (SD) | Median | Mean (SD) | Median | Mean (SD) |
| Gender |  |  |  |  |  |  |  |  |  |  |  |
| Male | 60 | 15.0 | 20.3 (17.4) | 5.5 | 5.59 (3.5) | 4.0 | 5.1 (4.6) | 3.0 | 5.2 (5.8) | 2.0 | 4.1 (6.3) |
| Female | 60 | 19.0 | 27.6 (25.2) | 5.5 | 7.0 (6.7) | 4.0 | 6.3 (6.3) | 6.5 | 8.5 (8.5) | 3.0 | 5.7 (7.7) |
| Age |  |  |  |  |  |  |  |  |  |  |  |
| 11 | 30 | 17.5 | 26.9 (25.2) | 7.5 | 7.4 (4.1)* | 4.0 | 5.3 (4.7) | 4.5 | 7.6 (8.4) | 3.0 | 6.5 (9.7) |
| 12 | 30 | 23.5 | 28.7 (22.9) | 6.0 | 8.2 (8.2) | 5.5 | 7.1 (6.0) | 4.5 | 8.1 (7.7) | 4.0 | 5.3 (5.8) |
| 13 | 30 | 10.5 | 19.3 (21.1) | 4.0 | 5.4 (3.9) | 3.5 | 4.7 (5.5) | 2.0 | 4.9 (7.4) | 1.0 | 4.2 (6.9) |
| 14 | 30 | 15.0 | 20.7 (17.0) | 4.0 | 4.7 (3.0)* | 3.5 | 5.7 (5.8) | 5.0 | 6.8 (6.0) | 2.0 | 3.5 (4.6) |
| Dental caries |  |  |  |  |  |  |  |  |  |  |  |
| DMFT = 0 | 55 | 24.0 | 23.1 (23.1) | 8.5 | 6.1 (4.8) | 5.0 | 5.5 (5.6) | 6.0 | 6.9 (7.9) | 7.0 | 4.8 (7.4) |
| DMFT = 1 or 2 | 32 | 6.0 | 5.6 (2.8) | 6.0 | 5.6 (2.8) | 4.5 | 5.5 (4.6) | 4.0 | 6.8 (6.3) | 2.0 | 4.1 (5.9) |
| DMFT ≥ 3 | 32 | 17.0 | 26.9 (24.8) | 6.0 | 7.8 (7.6) | 4.0 | 6.3 (6.2) | 4.0 | 6.9 (7.8) | 3.0 | 5.9 (7.5) |
| dmft = 0 | 113 | 15.0 | 22.4 (20.5)* | 5.0 | 6.2 (5.4)* | 4.0 | 5.4 (5.2) | 4.0 | 6.4 (6.9)** | 2.0 | 4.3 (6.4)** |
| dmft ≥ 1 | 7 | 48.0 | 48.4 (30.5)* | 13.0 | 9.9 (3.8)* | 12.0 | 10.3 (8.0) | 12.0 | 15.0 (10.6)** | 11.0 | 14.0 (10.6)** |
| Fluorosis |  |  |  |  |  |  |  |  |  |  |  |
| 0 | 100 | 17.0 | 24.9 (22.4) | 6.0 | 6.7 (5.5) | 4.0 | 5.9 (5.8) | 4.0 | 7.1 (7.3) | 2.0 | 5.2 (7.5) |
| ≥ 1 | 20 | 14.0 | 19.0 (18.8) | 5.0 | 5.1 (4.1) | 4.0 | 4.7 (4.1) | 2.0 | 5.8 (8.3) | 2.0 | 3.4 (3.6) |
| Gingivitis |  |  |  |  |  |  |  |  |  |  |  |
| Absence | 74 | 15.0 | 21.2 (19.4) | 5.0 | 5.6 (3.7) | 3.5 | 5.1 (5.4) | 4.0 | 6.2 (6.6) | 2.0 | 4.3 (6.1) |
| Presence | 46 | 18.5 | 28.3 (25.0) | 6.0 | 7.9 (7.0) | 5.0 | 6.7 (5.6) | 4.0 | 7.9 (8.5) | 2.5 | 5.9 (8.3) |
| Malocclusion |  |  |  |  |  |  |  |  |  |  |  |
| Minor/none | 81 | 15.0 | 20.9 (19.5) | 5.0 | 5.7 (3.5) | 4.0 | 5.0 (4.9) | 3.0 | 5.9 (7.1) | 2.0 | 4.2 (6.4) |
| Definitive | 18 | 24.0 | 32.1 (24.8) | 7.0 | 9.7 (9.8) | 6.0 | 8.1 (7.4) | 7.0 | 8.8 (7.1) | 4.5 | 5.6 (5.6) |
| Severe | 9 | 22.0 | 37.0 (34.0) | 4.0 | 8.1 (7.1) | 9.0 | 7.9 (6.6) | 9.0 | 11.2 (10.6) | 6.0 | 9.8 (13.2) |
| Handicapping | 12 | 17.5 | 21.9 (17.5) | 5.5 | 5.4 (2.6) | 4.0 | 5.1 (4.6) | 7.0 | 6.8 (6.4) | 2.5 | 4.6 (6.4) |

*p≤0.05 **p≤0.01
